# Hybrid architecture based intelligent diagnosis assistant for GP

**DOI:** 10.1186/s12911-023-02398-8

**Published:** 2024-01-10

**Authors:** Ruibin Wang, Kavisha Jayathunge, Rupert Page, Hailing Li, Jian Jun Zhang, Xiaosong Yang

**Affiliations:** 1 National Centre for Computer Animation, Bournemouth University, Bournemouth, UK; 2 Poole Hospital NHS Foundation Trust, Poole, UK; 3 Animation and Digital Art, Communication University of China, Beijing, China

**Keywords:** GP, Referral letter, Primary diagnosis, Hybrid architecture, Text classification, AI diagnosis assistant

## Abstract

As the first point of contact for patients, General Practitioners (GPs) play a crucial role in the National Health Service (NHS). An accurate primary diagnosis from the GP can alleviate the burden on specialists and reduce the time needed to re-confirm the patient’s condition, allowing for more efficient further examinations. However, GPs have broad but less specialized knowledge, which limits the accuracy of their diagnosis. Therefore, it is imperative to introduce an intelligent system to assist GPs in making decisions. This paper introduces two data augmentation methods, the Complaint Symptoms Integration Method and Symptom Dot Separating Method, to integrate essential information into the Integration dataset. Additionally, it proposes a hybrid architecture that fuses the features of words from different representation spaces. Experiments demonstrate that, compared to commonly used pre-trained attention-based models, our hybrid architecture delivers the best classification performance for four common neurological diseases on the enhanced Integration dataset. For example, the classification accuracy of the BERT+CNN hybrid architecture is 0.897, which is a 5.1% improvement over both BERT and CNN with 0.846. Finally, this paper develops an AI diagnosis assistant web application that leverages the superior performance of this architecture to help GPs complete primary diagnosis efficiently and accurately.

## Background

In many healthcare systems, including the United Kingdom and Ireland, General Practitioners (GPs) are the first point of contact for patients and most general problems are addressed in primary care [1]. GP plays a central role in ensuring that the patient receives a timely and preliminary diagnosis. However, if GP is unable to make decisions, the patient will be referred to a specialist.

For a patient eligible for referral, GP will write a referral letter to a specialist, outlining the reason why this patient is being referred, as well as the patient’s past medical history, including medications, allergies and any other relevant information. After 2 weeks (urgent situations) or 18 weeks (non-urgent) wait, an appointment with a hospital specialist will be offered to the patient.

With the outbreak of COVID-19, the NHS is under extreme pressure. Hospital Episode Statistics (HES) data shows that the number of unsuccessful GP referrals has jumped from 238,859 in February 2020 to a staggering 401,115 in November 2021 (an 87% increase), because there is no capacity in secondary care, and those referrals are rejected. During the specialist consultation process, almost two-thirds of the time is spent on confirming the contents of the referral letter and doing basic examinations, then they upgrade to the professional tests and make a decision. If this initial confirmation and basic examination process are completed before the specialist consultation, this process will be accelerated which can save a lot of time and more patients will have a chance to get health care. GP is the most suitable role to take over this work. As the first point of contact for patients, GP has the best understanding of the patient’s situation. Because GP has a broader, less specialized knowledge base, the accuracy of their primary diagnosis is limited. If there is an assistant for the GP to improve the accuracy of their primary diagnosis, it will be helpful to relieve the pressure on the specialist. A lot of research has been devoted to developing AI-assisted diagnosis methods [2–5] or tools (i.e., Symptomate, Patient, HealthTap, Ada and etc.). However, they are mainly facing the patient, and the connection between GP and specialists is seldom considered.

Therefore, the purpose of the paper is to develop an intelligent GP assistant which can improve the accuracy of the GP’s primary diagnosis. As the referral letter includes abundant information about the patient’s past medical history including medications, allergies and any other relevant information. The proposed system takes the complaints in the referral letter and the final diagnosis from specialists to train an AI model. We collaborate with neurologists at the University Hospital Dorset (UHD) to collect the source datasets. The proposed architecture supports the diagnosis of 4 common neurological diseases including epilepsy-recurrent seizures, headache, dorsalgia and cerebral infarction.

Diagnosing on the basis of a referral letter can be seen as the text classification task. With the development of the research in natural language processing, there are mainly two types of models that can be used to complete this task: the traditional deep learning-based text classification models including Convolutional Neural Network (CNN) [6, 7], Long Short-Term Memory networks (LSTM) [8] and their variants; the pre-trained attention-based models including Transfomer [9], Bidirectional Encoder Representations from Transformers (BERT) [10], DistilBERT [11], XLNet [12] and Robustly Optimized BERT approach (Roberta) [13].

Distinct from the typical text classification task, the symptoms present in the content for classification will play a pivotal role in determining the final outcome. Our dataset not only contains the long complaint text but also includes short symptom phrases (extracted symptoms). For the traditional deep learning-based model, the feature of each word uses static (fixed) word vectors that come from the pre-trained word embedding models, they have a better performance in short text classification. As embedding models themselves, the pre-trained attention-based structures have the advantage of extracting the long-distance dependencies in the text by using a multi-head attention approach to calculate the similarities between words. Therefore, fusing them together can not only enrich the representation of our neurology dataset but also can benefit from both short-text symptoms and long-text complaints.

A hybrid architecture is proposed in this paper, which fuses the features of words in different representation spaces to make the primary diagnosis from a referral letter. This structure jointly optimizes the pre-trained attention-based model and traditional deep learning model, which makes full use of the two different representation spaces. Besides, two data augmentation methods (Complaint-Symptoms Integration Method and Symptom Dot Separating Method) have also been proposed for this architecture. Subsequent experiments demonstrate that this hybrid architecture has a better performance on the accuracy of the text classification task. Finally, we develop an AI diagnosis assistant web application which leverages the superior performance of this architecture to help GPs complete referral tasks more efficiently and accurately. Our code is available at https://github.com/ruibin-wang/referral-letter-classification.

The contribution of this paper can be summarized as follows:

- This paper proposed a **Hybrid Architecture** which fuses the features of words in different representation spaces.
- This paper proposed a **Symptom Dot Separating Method** to avoid semantic confusion of the extracted symptoms.
- A **Complaint-Symptoms Integration Method** is proposed which is proven to have a positive effect on the accuracy of the disease classification tasks.
- An AI diagnosis assistant **web application** is developed to help GPs complete primary diagnosis more efficiently and accurately.

## Related work

The referral letters, encompassing detailed records of patients’ historical illnesses and current symptomatic presentations, offer a valuable resource for assisting GPs in decision-making. This study introduces a novel approach, utilizing referral letters as a predictive tool for disease diagnosis. To our knowledge, this is the first initiative to employ referral letters for disease diagnosis. Currently, the primary methodologies for aiding diagnosis include task-oriented dialogue systems and text classification methods. This proposed use of referral letters aims to complement these existing techniques, potentially enhancing the diagnostic process.

### The task-oriented dialogue systems

In the realm of task-oriented methodologies, as referenced in studies [2–5], the primary focus lies on developing a coherent dialogue policy. This policy aims to elicit detailed information from patients through conversation to facilitate accurate diagnoses. The foundational data in these methods is derived from dialogues between patients and doctors. It is important to note that the nature of this source data differs from the focus of our current research problem.

### Text classification methods

The complaint in the referral letter presents the patient’s medical information with a long text. Text classification methods can be used for mining useful information in referral letters and classifying it into the correct disease categories. There are three main text classification methods: machine learning-based methods, traditional deep learning-based methods and pre-trained attention-based methods.

**Machine learning-based methods**: Machine learning approaches in this domain primarily relied on predefined features, such as associated symptoms and disease status, as inputs. These methods employed classifiers like Support Vector Machine (SVM) [14], tree-based methods [15–20], Logistic Regression [21], and Naive Bayes (NB) [22] for classification purposes. However, these approaches, deeply rooted in statistical theory, often necessitated extensive feature engineering. Such reliance on manually crafted features could overlook scenarios not encompassed in the predefined sets. This limitation renders these traditional methods less effective for the classification of referral letters, where the range of features might not be counted.

**Traditional deep learning-based methods**: The advent of word embedding techniques, as referenced in [14, 22–25], has significantly enhanced the efficacy of traditional deep learning-based methods in text classification tasks. These methods, including Convolutional Neural Networks (CNN) [6, 7], Long Short-Term Memory networks (LSTM) [8], and their various adaptations [7, 26, 27], leverage word embeddings to encode textual semantics into a word representation space. In these approaches, each word in the text is represented by static word vectors derived from pre-trained word embedding models such as fastText [16], word2vec [17–19], and GloVe [20]. The unique filter design of CNN and the attention mechanisms within LSTM make these models particularly adept at identifying local and positionally invariant features in texts, such as key phrases or terms crucial for classification. Despite this, the architectural constraints of CNN and LSTM, coupled with the use of static word embeddings, limit their effectiveness in classifying longer texts. This limitation arises from the inherent inability of these models to dynamically adapt word representations based on the surrounding textual context, which is often essential for understanding more extended text passages.

**Fine-tuning on the pre-trained attention-based methods**: Word representation varies across different semantic spaces, a concept exemplified by pre-trained attention-based models like Transfomer [9], BERT [10], DistilBERT [11], XLNet [12], Roberta [13]. These embedding models, trained on extensive datasets, offer rich contextual representations of words. Additionally, the incorporation of multi-head self-attention mechanisms within their architecture enables them to effectively handle long texts with complex dependencies. This capability is crucial for accurate text classification, as it allows for a more nuanced understanding of the text’s semantic structure.

In this research, we focus on a dataset of referral letters, where the key symptoms detailed within the text are crucial for classification outcomes. Additionally, the dependency attributes of these symptoms play a significant role in determining the final classification results. Therefore, it is essential to propose a method that can simultaneously consider both the overall context and the local key information within the text. In our discussion on text classification methodologies, CNN and LSTM techniques excel in recognizing local and positionally invariant features in text. However, their efficacy diminishes when dealing with extended text sequences. In contrast, pre-trained attention-based models show superior performance with longer texts but lack the precision to consistently highlight all key symptoms in the context. Therefore, we propose a hybrid architecture that involves an integration of these two model types. This hybrid architecture leverages the strengths of each model type, enhancing the system’s capacity for accurate text interpretation and classification. Complemented by appropriate data augmentation methods designed to emphasize key symptoms within the referral letters, the resultant architecture benefits from the synergistic combination, leading to a more robust and nuanced performance in text analysis. The subsequent section will detail the proposed data augmentation methods and the hybrid architecture.

## Methods

This paper proposed two data augmentation methods and a hybrid architecture which will be introduced in this section.

### Data pre-processing

**Fig. 1:**
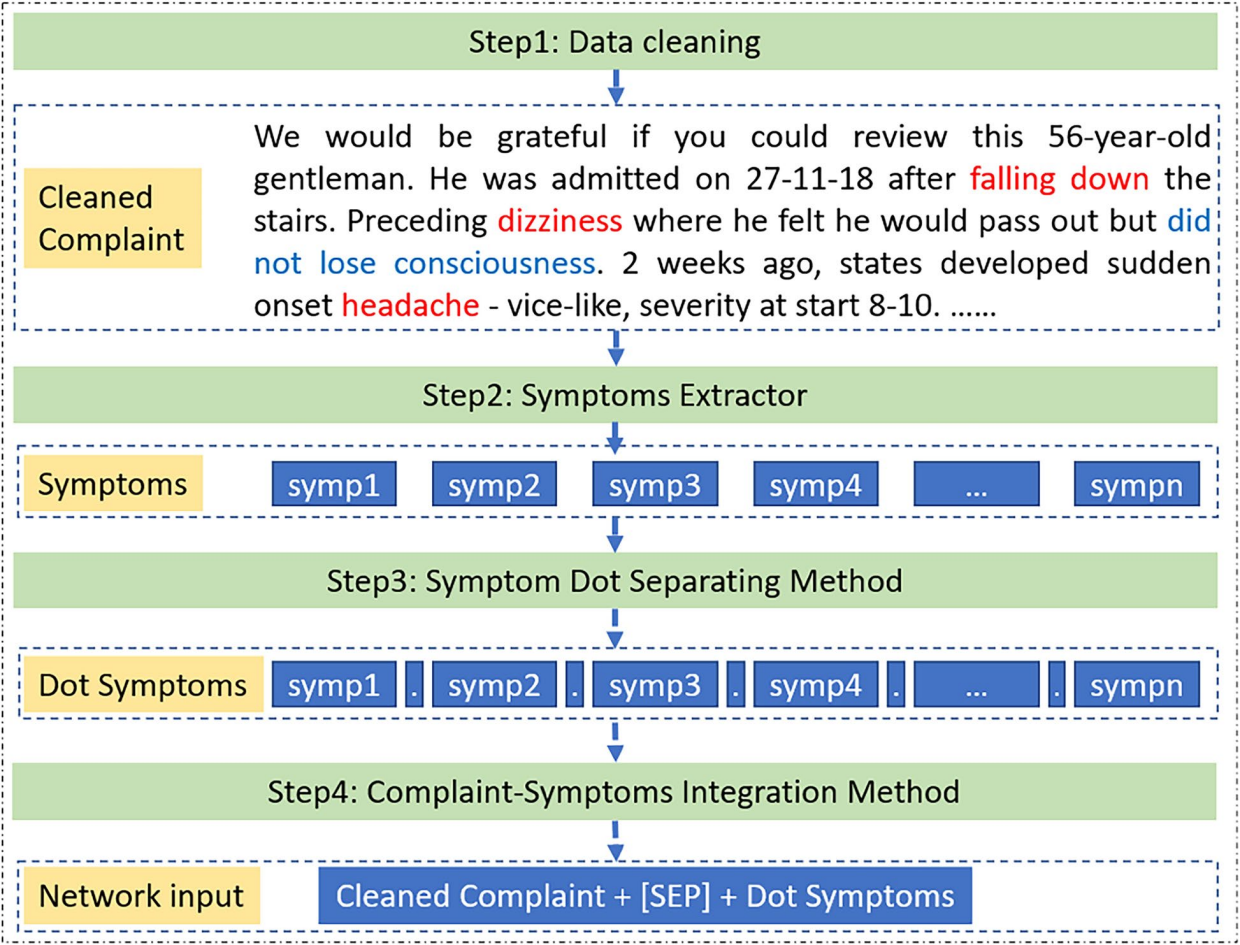
Flow chart of the data processing

This paper collected a dataset of referral letters from neurologists at UHD. Our dataset includes records of four common neurological diseases: epilepsy-recurrent seizures (G40), headache (R51), dorsalgia (M54), and cerebral infarction (I63), along with their corresponding complaint texts. An example of this dataset can be found in Table 1, and the dataset’s statistics are presented in Table 2. In Tables 1 and  2, the referral letter presents the patient’s clinical condition and medical history information in the complaint text. The codes G40, R51, I63, and M54 correspond to the International Classification of Disease (ICD) codes. As shown in Fig. 1, before training the hybrid architecture, the complaint text in the referral letter is processed with the following four steps.

**Table 1:** Referral letter examples for four diseases

Complaint text	Diagnosis
We would be grateful if you could review this 56-year-old gentleman. He was admitted on 27-11-18 after falling down the stairs. Preceding dizziness where he felt he would pass out but did not lose consciousness. 2 weeks ago, states developed sudden onset headache - vice-like, severity at start 8-10. Not positional. Right eyes have tunnel vision. On examination had reduced abduction right eye and diplopia. The case was discussed with Dr xxx who suggested MRI. The MRI report is as below and suggests a neurology review.	R51, Headache
Mrs XXXX was admitted with new left arm weakness on a background of muscular dystrophy. A CT showed no intracranial acute abnormality. There was an unexpected finding of bifrontal disproportionate apparent volume loss, with a lack of transverse veins which could represent inconspicuous subdural haematomas - for which a follow-up CT scan in 2 weeks has been requested. Our impression is of muscular dystrophy progression.	I63, Cerebral infarction
History of leg pain and numbness. This lady related a history of 2 years or so of right leg numbness secondary to chronic back pain. She related a 2-week history of left-sided numbness in the thighs and worsening of her back pain. Examination reveals that she has decreased sensation in the left L4-5 nerve route distribution on the medial aspect of the left calf and dorsal aspect of the left foot. The plantar sensation is normal. Power to L4-5 and S1 is normal although she is unable to flex her hips fully and cannot perform an active SLR.	M54, Dorsalgia
We would be grateful for your assessment of this 47-year-old lady. She has known Epilepsy post-head-injury ten years ago and has not had a seizure in 3 years. She presented last week with 3 x tonic-colonic seizures, each of which terminated with Diazepam. She usually takes Carbamazepine 400mg BD, however, had not been taking this for the previous few weeks. She still has a residual left-sided weakness. Power in her left leg remains at 1-2-5, and the left arm-hand at 2-3-5. I discussed her with the on-call neurologist on Friday who advised starting her back on her normal medication with 7-10 days of Clobazam to cover. She is still very fatigued and sleeping much of the day.	G40, Epilepsy-recurrent seizures

**Table 2:** Statistic of the neurology dataset

Disease code	Number of referral letters
G40	79
R51	65
M54	59
I63	58
**Total**	261

Step1: Data cleaning

The data source utilized in this study comprises referral letters, typically formatted as emails, written by GPs addressing specialists. Upon examining the referral letters, it was observed that a minor portion of the data contained special characters, such as “$$", “**", “Å", “??" and “XXX", in addition to standard greeting phrases. These elements, unrelated to clinical content, have the potential to obscure critical information within the text. The removal of such characters and words is crucial for diminishing dataset noise and enhancing the focus on pertinent information, which is anticipated to improve the accuracy of disease diagnosis. This paper employs character-matching techniques to systematically eliminate these irrelevant elements.

Step2: Extract symptoms

Symptoms are the key information for a GP to make decisions. A clear and abundant description of symptoms will improve the accuracy of diagnosis. Therefore, this step is to extract symptoms in the given text. Although MIE [28], BERN [29] and Stanza [30] can recognize some symptoms, the accuracy is not satisfactory, and negated symptoms cannot be recognized. This paper adopts the Watson Annotator for Clinical Data of IBM^1^, which can provide a high recognition accuracy for symptoms. Negated symptoms are symptoms that the patient does not have, but which have been mentioned in the complaint text, they will be removed in this step, for example, the blue text in Table 3.

Step3: Symptom Dot Separating Method

Most symptoms consist of multi-words such as falling down, pass out and tunnel vision in Table 3. If these symptoms are connected directly in a sequence as the input for models, the relation between connection words (for example the word ‘down’ and ‘dizziness’ in the processed symptoms part of Table 3) will be calculated, which will extract confusing information from the sequence and has a negative effect on the accuracy of disease classification. Therefore, this paper proposed a **Symptom Dot Separating Method**, using an unmeaningful symbol dot (‘.’) to integrate the extracted symptoms into training, validating and testing data. Lateral experiments will prove that under the same network structure, the classification performance of training data processed with the Symptom Dot Separating Method is better than that without using this method. The processed symptoms can be seen in Table 3.

Step4: Complaint-Symptoms Integration Method

As described in the Related work section, to effectively harness the potential of referral letters in assisting GPs with decision-making, it is essential to employ text classification methods. For machine learning approaches, predefined features (such as related symptoms or fitness states) are essential for disease diagnosis. Task-oriented dialogue systems [2–5], aim to develop effective dialogue policies to gather symptom information from patients, employing predefined features as slots to be filled based on conversation content. This indicates the pivotal role of symptoms or fitness states in diagnosis processes. However, defining these features beforehand is labour-intensive and has difficulty addressing complex situations. Employing Transformer-based models leverages their capability to handle long dependencies, thereby obviating the need for labour-intensive feature definition. This allows for the direct use of extensive descriptive text in disease diagnosis, as referenced in [31]. However, a limitation of these Transformer-based approaches is their equal treatment of each word, which may lead to a diminished focus on disease-specific information within the text.

This paper proposes a **Complaint- Symptoms Integration Method**, which combines the complaint text and processed symptoms with a simple symbol “[SEP]". This symbol is often used in pre-trained attention-based models as a special connection between two different sentences. Our integration method not only keeps the original semantics of the patient’s historical medical information but also highlights key information in the complaint text. Subsequent experiments have shown that the classification accuracy of this method is higher than that of training complaint text and extracting symptoms alone.

**Table 3:** Example of complaint text processing

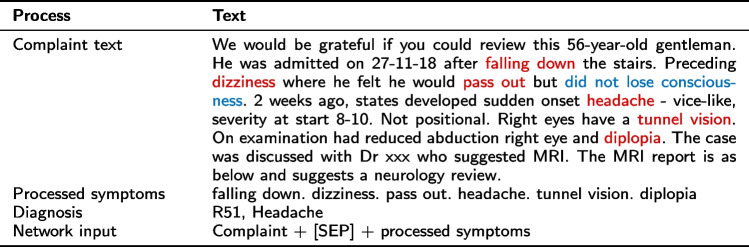	

Table 3 shows an example of complaint text processing. It can be clearly seen that the related symptoms are correctly extracted (marked in red in the complaint text), while negated symptoms are also obtained (marked in blue) and not included in the processed symptoms. Besides, three datasets **Symptoms**, **Complaints**, and **Integrations** are generated to validate the performance of our proposed methods and architectures. The Symptoms dataset only contains the symptoms processed by Step3 (for example processed symptoms in Table 3). The Complaints dataset only contains the cleaned complaint text processed by Step1(for example complaint text in Table 3). The Integration dataset consists of data generated through Step4 (for example network input in Table 3).

### The hybrid architecture

Our architecture integrates a pre-trained attention-based model (such as BERT, DistilBERT, XLNet, or Roberta) with a traditional deep learning model (like CNN, LSTM, or their variants). This hybrid approach leverages the strengths of both model types. Pre-trained models, having been extensively trained on large datasets, are adept at comprehending complex language and semantic nuances. They provide a context-rich word representation, which is vital for precise text classification. Meanwhile, the CNN or LSTM models are particularly effective in identifying local and positionally invariant features in text, like key phrases or terms that are critical for classification. By merging these two model types, our architecture benefits from their complementary capabilities. This synergy enhances the system’s ability to interpret and classify text, yielding a more robust and nuanced performance.

**Fig. 2:**
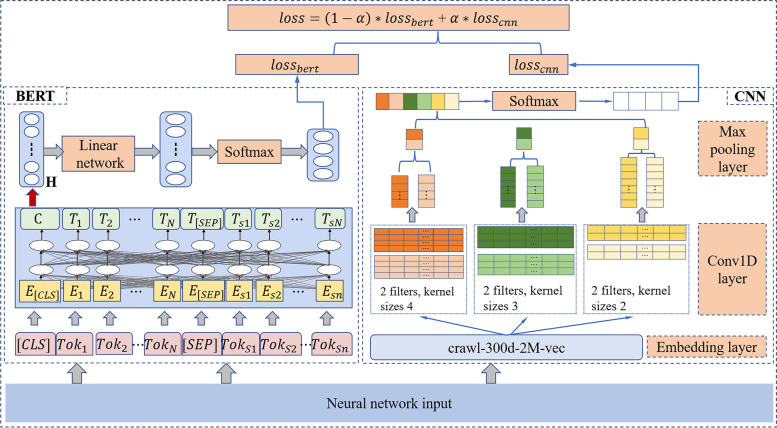
The hybrid architecture

The input of this architecture is the three datasets processed in “Data pre-processing” section and the output is the prediction of the 4 common neurological diseases. We take BERT+CNN as an example to explain the training process. As depicted in Fig. 2, the left part is the BERT branch, and the right part is the CNN branch (the Bert branch can be changed to other pre-trained attention-based models).

Given the neural network input data as $X=(x_1,...x_i,...,x_m)$, four diseases as $Y=(y_1,...,y_4)$ in which $x_i$ is the *ith* word in the text and *m* is the length of the text.

BERT Branch

The first hidden state H in Fig. 2 of the BERT model presents the features of the whole input text, it is used as the input of the lateral linear layer. This paper utilizes the sigmoid function in the softmax layer as

1 $$\begin{aligned} Bert_{logits} = Softmax(Linear(H)). \end{aligned}$$

The Cross-Entropy Loss is used as our loss function, the loss of the BERT branch is described as

2 $$\begin{aligned} Loss_{bert} = CrossEntropyLoss(Bert_{logits},Y). \end{aligned}$$

CNN Branch

Word embedding is a learned representation for text where words that have the same meaning would have a similar representation. As mentioned in the introduction part, there are mainly four pre-trained word embedding models, because of the wide coverage of different semantic lexical, the ‘crawl-300d-2M.vec’ [32] (2-million-word vectors trained on Common Crawl (600B tokens)) of the Google’s fastText are utilized as the embedding layer of CNN as shown in Eq. (3).

3 $$\begin{aligned} X_{vector} =Embedding(X). \end{aligned}$$

The 1-dimension CNN is chosen as the convolutional layer, which is shown below:

4 $$\begin{aligned} CNN_{output} = conv1d(X_{vector}). \end{aligned}$$

The max pooling layer and linear classification layer are shown in Eq. (5).

5 $$\begin{aligned} CNN_{linear}=Linear(Maxpooling(CNN_{output})). \end{aligned}$$

The softmax layer is described as following

6 $$\begin{aligned} CNN_{logist} = Softmax(CNN_{linear}). \end{aligned}$$

The loss function of the CNN model is the same as BERT, as shown

7 $$\begin{aligned} Loss_{CNN}= CrossEntropyLoss(CNN_{logist},Y). \end{aligned}$$

A parameter $\alpha$ for the hybrid architecture is used to fuse the two branches, presented below

8 $$\begin{aligned} Loss = \alpha *Loss_{CNN}+ (1-\alpha )*Loss_{Bert}. \end{aligned}$$

The $(m+1)th$ fusion weight $\alpha _{m+1}$ is updated by the following equation

9 $$\begin{aligned} \alpha _{m+1}=\alpha _m-(Loss_{CNN}-Loss_{Bert})*lr*\alpha _m. \end{aligned}$$

In which the parameter *lr* is the learning rate.

## Experiment

### Experiment settings

This paper prepares three training datasets, namely the Symptoms dataset, Complaints dataset and Integration dataset to validate the performance of our proposed architecture and methods. The Symptoms dataset and Complaints dataset consists of processed symptoms and complaints, respectively, and the Integration dataset consists of texts processed by the Complaint-Symptoms Integration Method. To validate the hybrid architecture and the proposed training strategies, the training, validation and testing samples are divided in a ratio of 7:2:1, which have 179/51/27 samples, respectively. Meanwhile, four tasks are arranged using classification accuracy as the metric.

### Bert branch baseline

Comparing the performance of different pre-trained attention-based models on our proposed hybrid architecture, four commonly used sentence classification models are adopted. The details of the four models are as below:

- **BERT** is a pre-trained language model that has achieved state-of-the-art performance in various natural language processing (NLP) tasks. It uses a bidirectional transformer architecture, which allows it to consider the entire context of a sentence or text when generating its representations.
- **DistilBERT** is a smaller and faster version of BERT, designed for resource-constrained environments, while still maintaining high accuracy in various NLP tasks. It uses a distillation technique to transfer the knowledge from the larger BERT model to a smaller one.
- **XLNet** is a transformer-based language model that aims to address some of the limitations of previous models like BERT. It uses a permutation-based approach for generating representations, which allows it to capture more diverse and complex relationships between words in a sentence.
- **RoBERTa** (Robustly Optimized BERT approach) is another variant of BERT, which was pre-trained on a larger dataset and with improved training techniques. It has achieved state-of-the-art performance on several NLP tasks, outperforming BERT and other previous models.

**Parameters setting**: The batch size is set to 8, dropout $p=0.5$, the optimization method is Adam [33] and the learning rate is set as 1e-6. For the CNN branch, this paper chooses the 1-dimension CNN which has three kernel sizes (2,3,4) and each kernel has two filters. Besides, the initial value of $\alpha$ for the hybrid architecture is set as 0.3 with lr=1e-5. The hidden unit of CNN uses the fixed embedding representation vector (dimension: 300) and the epoch is set as 200.

The configuration of the computer is Windows 10, Nvidia RTX 2080super, Intel Core (TM) i7-9700 and RAM 32G.

### Experimental design

In order to validate the diagnostic accuracy of our proposed methods, three experiments are designed. Besides, three training methods are adopted for each architecture, including training on the symptoms processed with the Symptom Dot Separating Method, training on the complaint text only and training on the data processed with the Complaint-Symptoms Integration Method.

**Fine-tuning the pre-trained attention-based model**: This step will further fine-tune the pre-trained attention-based models such as BERT, DistilBERT, XLNet and Roberta on the neurology referral letter dataset. The architecture of this step can be found in the left BERT branch of Fig. 2.

**The hybrid architecture**: This part is designed to compare the accuracy of different combinations between BERT+CNN, DistilBERT+CNN, Roberta+CNN and XLNet+CNN to validate the performance of the proposed hybrid architecture.

**Fig. 3:**
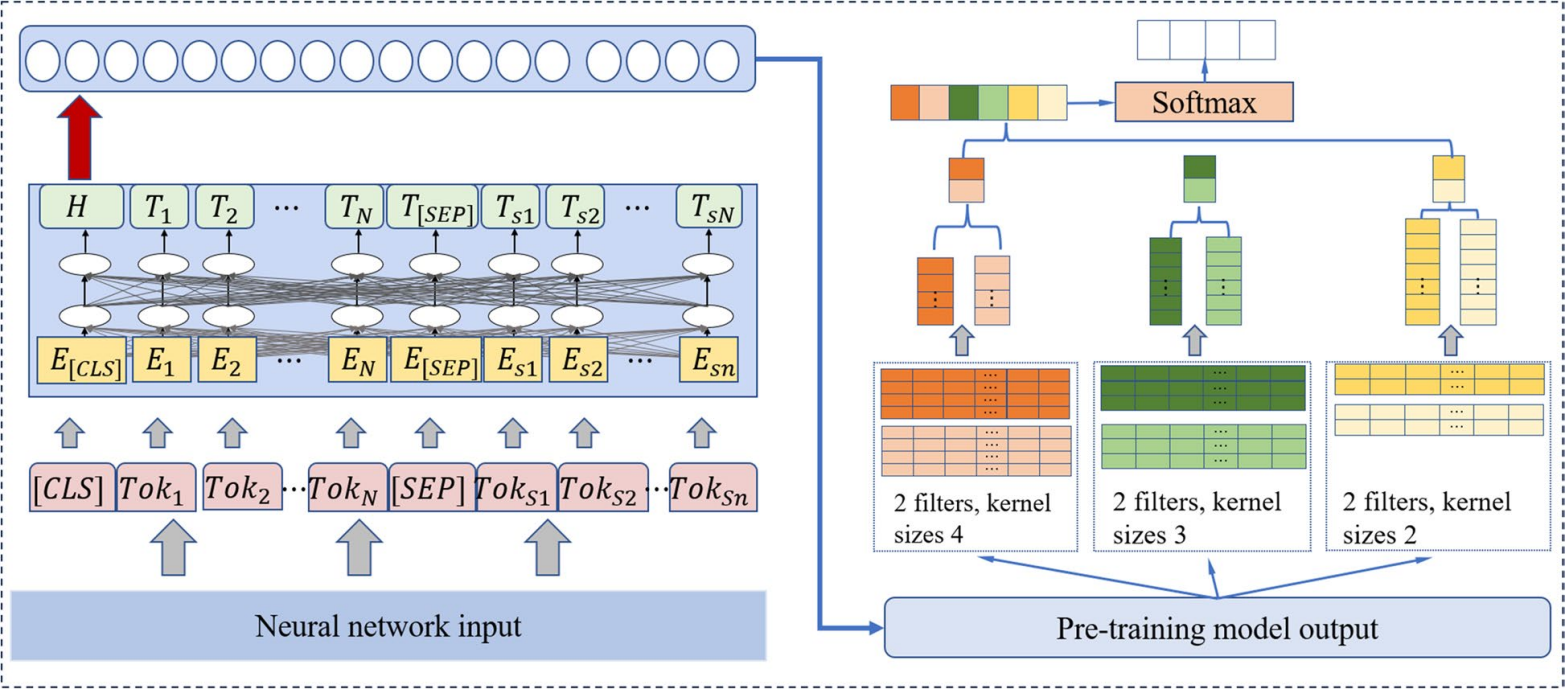
Bert-CNN sequential model

**Comparison with the sequential model**: As shown in Fig. 3, the sequential model consists of the pre-trained attention-based network and CNN network, and the pre-trained attention-based model can be seen as the embedding of CNN. A single embedding representation space is utilized in this architecture, and it is used to compare the effectiveness of the hybrid architecture which fuses two different embedding representation spaces. Four models are designed for this task, including the BERT-CNN, DistilBERT-CNN, Roberta-CNN and XLNet-CNN. The hidden unit of CNN in this part keeps the same dimension as the pre-trained attention-based embedding model (768).

**Validation of adding dot**: By comparing the performance of adding dots and without dots for the extracted symptoms, this task is designed to validate the effect of the Symptom Dot Separating Method on our proposed hybrid architecture.

### Main results

Results of the design tasks on our neurology dataset are presented in Table 4. As described in “Experimental design” section, the first vertical column of Table 4 shows three designed architectures, and the second column shows different models in each architecture. Symptoms, Complaints and Integration are three training datasets described in “Data pre-processing” section.

**Table 4:** Results of the four designed experiments

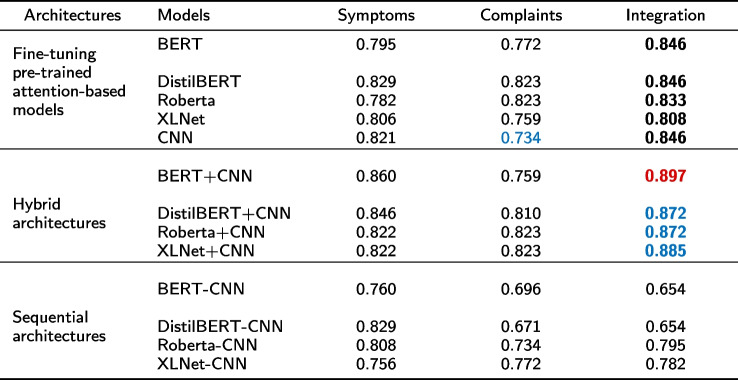	

Note: The use of the special fonts (red, blue, and bold) in the table is intended to assist readers in better understanding the comparison of relevant experiments in the subsequent content

As shown in the black bold data of pre-trained attention-based models in Table 4, the classification accuracy of training on the Integration dataset for each model is significantly higher than the results of training on Complaints and extracted Symptoms alone. With an average of 3% and 3.9% improvement on the Symptoms and Complaints datasets respectively. Besides, CNN also has an obvious improvement. It indicates that training on the data processed with our data augmentation methods will produce better classification accuracy. Comparing the result in the Complaints column of pre-trained models with CNN, it can be found that fine-tuning on pre-trained attention-based models perform better than CNN (as 0.734 is lower than the other results in the complaints column of Table 4 and highlighted in blue). But our proposed Complaint-Symptoms Integrating training method ‘re-energizing’ CNN, makes it outperform Roberta and XLNet and achieve comparable performance to BERT and DistilBERT. This also can be evidence of the positive effect of our proposed data integration method.

Two conclusions can be drawn from the hybrid architecture of Table 4:

1. By comparing the performance of hybrid architectures and their associated pre-trained attention-based models under the same training dataset (for example comparing BERT+CNN and BERT on the Symptoms, Complaints or Integration dataset), it can be found that on both Symptoms and Integration datasets, all hybrid architectures outperform pre-trained attention-based models. Besides, BERT+CNN architecture obtains the best classification accuracy on our integration dataset with a 5.1% improvement.

**Fig. 4:**
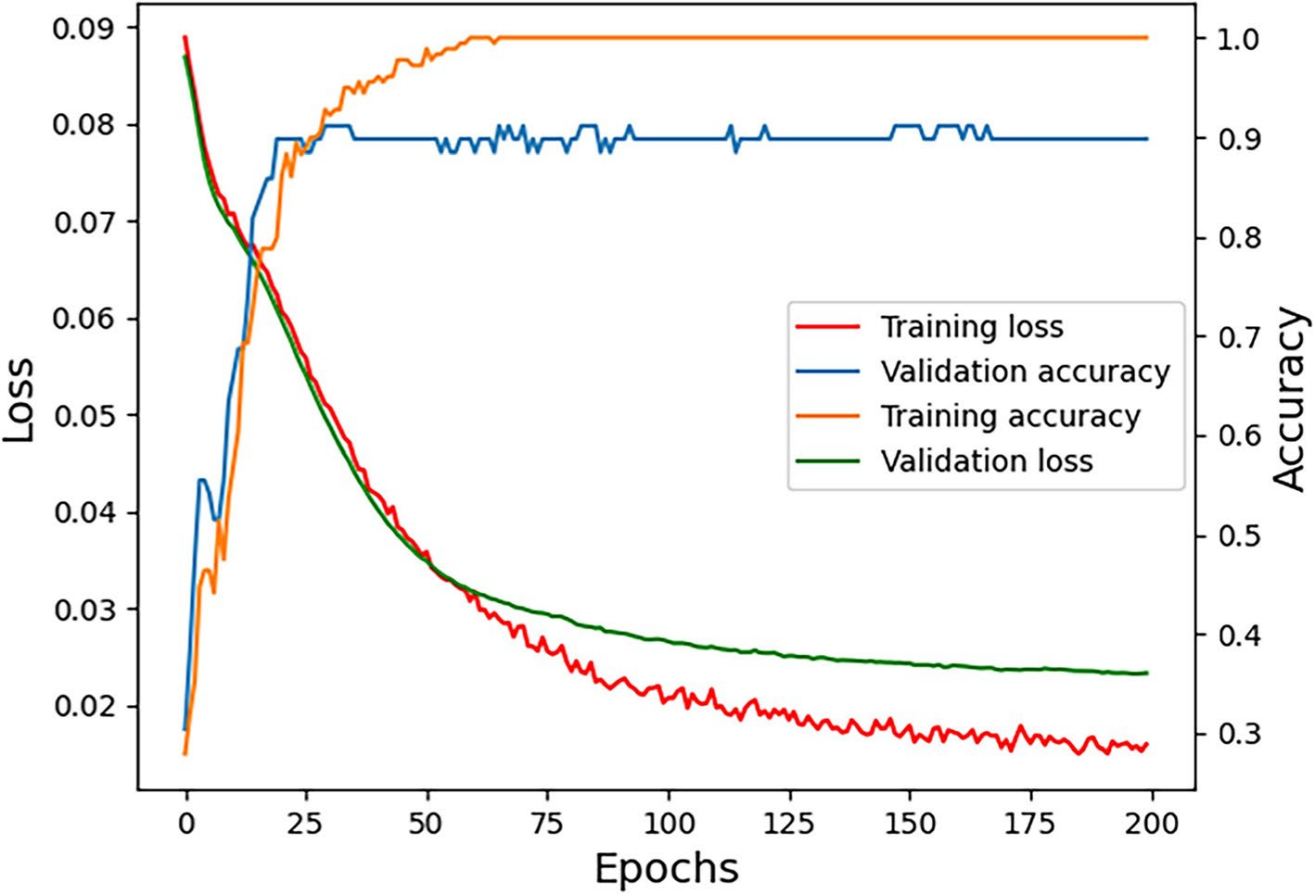
Training and validation loss and accuracy for the BERT+CNN architecture

To demonstrate the efficacy of the hybrid architecture, Fig. 4 provides a visual representation of the training and validation processes in terms of both loss and accuracy. Specifically, Fig. 4 showcases the performance metrics of the BERT+CNN architecture, serving as a representative example. This illustration aids in comprehending the architecture’s effectiveness during the training phase and its subsequent validation. For Fig. 4, the left vertical axis represents the loss, with the red line indicating training loss and the green line denoting validation loss. The right vertical axis is designated for accuracy, where the blue line represents validation accuracy and the orange line shows training accuracy. The horizontal axis corresponds to the number of training epochs. From the data in Fig. 4, the BERT+CNN model demonstrates robust performance, as indicated by the converging trends in loss and accuracy, without evidence of overfitting. Notably, around 75 epochs, the model appears to reach a plateau in its learning capacity. This is reflected by the stabilization of training accuracy, despite a continuing decrease in training loss. Consequently, the validation values exhibit fluctuations around this convergence point.

2. As for the hybrid architectures in Table 4, by comparing the classification accuracy of different training datasets under the same architecture, the blue bold data in Table 4 shows that all non-sequential architectures benefit from our Integration dataset: models trained on it consistently achieve better performance than either Symptoms or Complaints on their own.

These two conclusions demonstrate the positive effect of our hybrid architecture and two data augmentation methods on neurology disease classification tasks. Our method brings a maximum of 11% improvement for disease classification. The reasons for this improved performance are twofold: the two data augmentation methods enrich the content of training data, and they combined the original complaint text with the extracted symptoms. This highlights the key information in the complaint text. Another is that the hybrid architecture fuses two embedding spaces, which enriches the representation of our neurology dataset. Therefore, training on them results in better classification accuracy.

Comparing the classification accuracy of the sequential architectures and their related hybrid architectures (such as BERT+CNN and BERT-CNN) in Table 4, our proposed pre-trained attention-based models+CNN outperforms pre-trained attention-based models-CNN on all three training datasets. This is because our architecture fuses two different embedding representation spaces, which can improve the accuracy of neurological disease classification tasks significantly.

Due to the superior performance of the hybrid architecture BERT+CNN, it is chosen to validate the performance of the Symptom Dot Separating Method, the result can be found in Table 5. This table shows that adding dots between each symptom improves the classification accuracy of our proposed hybrid architecture. The main reason is that each symptom is separated, without semantic meaning, adding dots can avoid semantic confusion.

**Table 5:** Validating the performance of the Symptom Dot Separating Method

Conditions	Symptoms	Symptoms + Complaints
Without dot	0.829	0.846
With dot	**0.860**	**0.897**

This paper proposed three methods, including the hybrid architecture, the Symptom Dot Separating Method and the Complaint-Symptoms Integration Method which are proven to have positive effects on the accuracy of neurological disease classification tasks. This means that it can be a good assistant tool for GPs to make the primary diagnoses.

## Application

**Fig. 5:**
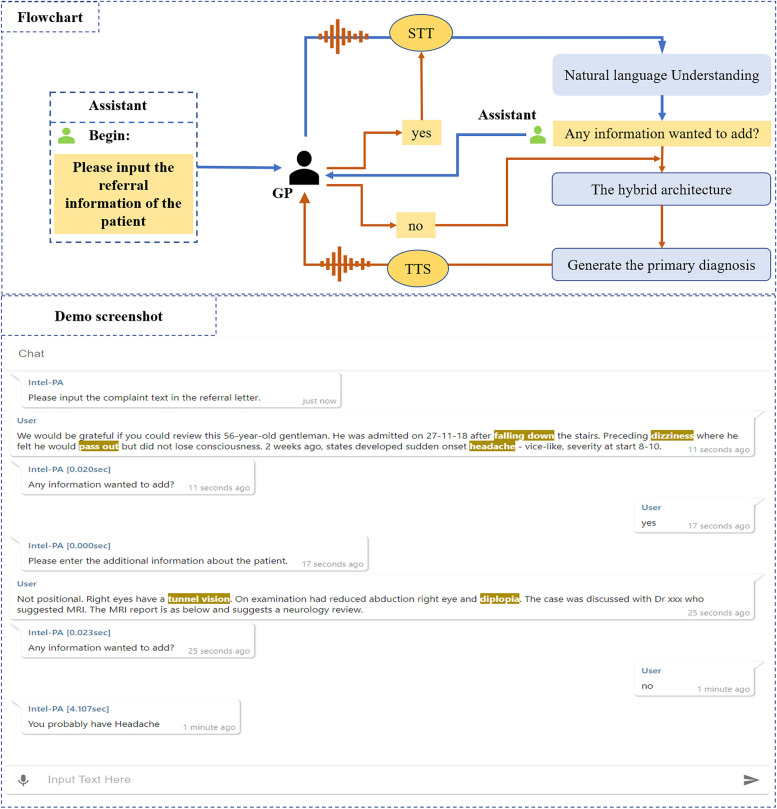
The flowchart and demo of the assistance diagnosis application which is developed on the hybrid architecture

In order to help GPs complete diagnosis more efficiently and accurately, this paper applied the hybrid architecture to develop an AI diagnosis assistant web application, which can help GPs make a primary diagnosis by talking/texting with them. Flowchart (left part in the figure) and demo screenshot of the conversation between a GP and AI diagnosis assistant (right part in the figure) can be found at Fig. 5. The natural language understanding part in Fig. 5 follows the same steps as the data processing in “Data pre-processing”. Besides, both Speech-to-text (STT) and Text-to-speech (TTS) functions are implemented using state-of-art methods, which can better improve the experience of this AI diagnosis assistant web application. A demonstration video which shows the process of both writing and speaking assistant process can be found at https://www.youtube.com/watch?v=Coj1xGYOCBw.

## Conclusion

This paper introduces a hybrid architecture to help GP to improve the accuracy of primary diagnosis. Comparative experiments on the neurology dataset demonstrate that our proposed model has satisfactory classification accuracy. We also proposed two data augmentation methods (the Symptom Dot Separating Method and the Complaint-Symptoms Integration Method) which were proved effective in improving the predicting accuracy of the neurology disease. This paper also applied the hybrid architecture to develop an AI diagnosis assistant web application, which can efficiently help GPs make a primary diagnosis by talking/texting with them. The accuracy of this architecture needs to be improved, and in the future, the relation between each symptom would be presented via the knowledge graph. The dataset and the source code can be found at the GitHub link which is shared in the Introduction section.

## Data Availability

The dataset utilized in the study is currently inaccessible as the collaborating doctor has no plan to share the data, citing complicated policies that must be followed for releasing a dataset. The sample data and source code can be found at the following link: https://github.com/ruibin-wang/referral-letter-classification.

## Footnotes

1 https://acd-try-it-out.mybluemix.net/
